# Incidence of postoperative facial weakness in parotid tumor surgery: a tumor subsite analysis of 794 parotidectomies

**DOI:** 10.1186/s12893-019-0666-6

**Published:** 2019-12-26

**Authors:** Hokyung Jin, Bo Young Kim, Heejung Kim, Eunkyu Lee, Woori Park, Sungyong Choi, Man Ki Chung, Young-Ik Son, Chung-Hwan Baek, Han-Sin Jeong

**Affiliations:** Department of Otorhinolaryngology - Head and Neck Surgery, Samsung Medical Center, Sungkyunkwan University School of Medicine, Seoul, Republic of Korea

**Keywords:** Parotid neoplasms, Surgical procedures, Postoperative complications, Facial nerve injuries, Treatment outcomes

## Abstract

**Background:**

The reported incidence of facial weakness immediately after parotid tumor surgery ranges from 14 to 65%. The purpose of this study was to evaluate the incidence of postoperative facial weakness related to parotidectomy with use of preoperative computed tomography (CT), intraoperative facial nerve monitoring, and surgical magnification. Also, we sought to elucidate additional information about risk factors for postoperative facial weakness in parotid tumor surgery, particularly focusing on the tumor subsites.

**Methods:**

We retrospectively reviewed 794 cases with parotidectomy for benign and malignant tumors arising from the parotid gland (2009–2016). Patients with pretreatment facial palsy were excluded from the analyses. Tumor subsites were stratified based on their anatomical relations to the facial nerve as superficial, deep, or both. Multivariable logistic regression analyses were conducted to identify risk factors for postoperative facial weakness.

**Results:**

The overall incidences of temporary and permanent (more than 6 months) facial weakness were 9.2 and 5.2% in our series utilizing preoperative CT, intraoperative facial nerve monitoring, and surgical magnification. Multivariable analysis revealed that old age, malignancy, and recurrent tumors (revision surgery) were common independent risk factors for both temporary and permanent postoperative facial weakness. In addition, tumor subsite (tumors involving superficial and deep lobe) was associated with postoperative facial weakness, but not tumor size. Extent of surgery was strongly correlated with tumor pathology (malignant tumors) and tumor subsite (tumors involving deep lobe).

**Conclusion:**

Aside from risk factors for facial weakness in parotid tumor surgery such as old age, malignant, or recurrent tumors, the location of tumors was found to be related to postoperative facial weakness. This study result may provide background data in a future prospective study and up-to-date information for patient counseling.

## Background

Functional preservation of the facial nerve in the affected gland is one of the most essential surgical steps in parotidectomy [1, 2]. To identify and preserve the facial nerve safely, many surgical landmarks have been proposed to locate the facial nerve consistently during parotidectomy [3–5]. In addition, preoperative imaging tests, intraoperative electromyographic monitoring of the facial nerve and use of a magnified surgical view (surgical loupes or microscope) have emerged as routine clinical steps in parotidectomy [6–13].

According to previous studies, 14 to 64% for temporary and 0 to 9% for permanent facial nerve weakness have been reported following parotidectomy [2, 10, 13–21]. Furthermore, much higher rates of facial nerve paresis were observed for revision parotid surgery [16, 22, 23], wide extent of surgery (total parotidectomy) [15, 16, 22–26], tumor located deeper than the facial nerve plane [17, 27], and in cases with large tumors [25, 28–30]. Recent studies also reported the improved rates of functional facial nerve preservation in parotidectomy, with the aid of preoperative imaging tests, intraoperative monitoring of the facial nerve, or surgical magnification [31–36]. Still there have been no strong evidence to support the preventive role of intra-operative facial nerve monitoring from facial nerve injury during parotidectomy. However, several studies suggested that rates of temporary or permanent facial palsy could be decreased in parotidectomy with intra-operative facial nerve monitoring [13, 14, 17, 25, 35].

In this study, we routinely applied preoperative computed tomography (CT) scans, intraoperative facial nerve monitoring, and surgical magnification for all parotidectomies, which is different from the previous studies. Preoperative CT scans is known to have a modest diagnostic accuracy for localization of parotid tumors in relation to the facial nerve using an imaginary facial nerve on images [37]. Thus, CT scans have some diagnostic limitations compared with the direct visualization of the facial nerve by advanced techniques of magnetic resonance imaging (MRI) [37]. However, preoperative CT could provide the gross characteristics of tumors in the parotid gland, approximate location of tumors, and sketchy surgical planning. More importantly, CT scans can be cost-effective and more popular in many countries, compared with the specialized MRI techniques. Combined with intraoperative facial nerve monitoring and surgical magnification, the surgeons may capture an advantageous position in functional preservation of the facial nerve during parotidectomy.

Thus, the purpose of this study was to evaluate the incidence of postoperative facial weakness related to parotidectomy with use of preoperative CT scans, intraoperative facial nerve monitoring, and surgical magnification. Also, we sought to identify risk factors for facial nerve palsy, particularly focusing on tumor subsites, which has been less studied. This study will provide background data in a future prospective study and up-to-date information for patient counseling.

## Methods

### Study subjects

This study was a retrospective analysis using clinical and pathological data from patients undergoing parotidectomy. The study protocol was approved by our Institutional Review Board (no. 2018–08-083) and the clinical data used in the study were de-identified.

The initial study population included 806 patients who had undergone parotidectomy for tumors arising in the parotid gland at our institution between 2009 and 2016. We included both benign and malignant tumors for analyses, as well as both primary surgery and revision parotid surgery for recurrent tumors. In some cases, the pathological diagnosis of malignant tumors were achieved after the detailed examination of surgical pathology. In addition, the extent of surgery could be overlapped between parotidectomy for benign tumor or low-grade malignant tumors [38, 39]. Thus, inclusion of both benign (majority) and malignant tumors (intact facial nerve) in the analyses seemed to be more realistic for patient counseling before surgery.

Among 806 patients, eleven patients who had preoperative facial weakness were excluded because we particularly focused on the occurrence of postoperative facial paralysis in this study. Another patient was also excluded from the study due to insufficient clinical information, leaving 794 eligible patients as the final number of subjects (Additional file 1: Raw data). Among patients with malignant parotid tumors without facial nerve weakness prior to surgery, some or all branches of the facial nerve were sacrificed for oncological safety in 21 patients, and these cases were included in the analysis.

### Preoperative imaging and intra-operative facial nerve monitoring

All subjects preoperatively underwent CT with contrast enhancement and fine needle aspiration cytology (ultrasonography-guided if indicated). CT scans were performed with non-contrast axial view (14.5 mm thickness), contrast-enhanced axial view (8 mm thickness) and contrast-enhanced coronal view (3.6 mm thickness). Additional imaging studies for tumor characterization and metastasis evaluation were conducted for patients with parotid tumors suspicious for malignancy (positron emission tomography and MRI imaging).

In this study, four surgeons (having more than 10 years of head and neck surgery experience, and treating more than 15 to 20 cases of parotidectomy every year) conducted the parotidectomies. No difference was found in terms of postoperative complications including facial weakness among responsible surgeons (data not shown). An intraoperative nerve monitoring system (four channel monitoring, NIM-Response 2.0, Medtronic, Minneapolis, MN, USA) was applied for facial nerve monitoring in all parotidectomy procedures, and the surgeons used a microscope or surgical loops to magnify surgical fields during parotidectomy. Tumor characteristics were determined by the pathological findings (tumor size and number). Tumor size was presented by the longest diameter of the tumor (cm), and in cases of multiple tumors, the sum of each tumor size (diameter) was calculated and regarded as tumor size. Tumor subsites were categorized into three compartments based on the surgical findings (anatomical relationship to the facial nerve) as follows: superficial to the facial nerve, deep to the facial nerve and tumors located in both the superficial and deep lobes crossing the facial nerve plane. The extent of surgery was divided into either total, superficial, partial parotidectomy, or extracapsular dissection of tumors. Total parotidectomy meant the removal of deep and superficial parotid lobes with facial nerve identification. Superficial parotidectomy was the surgical removal of the superficial parotid gland with facial nerve identification. On the other hand, partial parotidectomy was a resection of a part of the gland with identifying some of the facial nerve branches. Extracapsular dissection of tumors was a marginal removal of tumors without identification of the facial nerve [40–43].

### Evaluation of facial nerve function

The functional status of the facial nerve in the affected side was evaluated using the House-Brackmann grading system, [44] focusing on three subsites: forehead, eye, and lip. If a patient suffered from facial weakness in only one subsite, we regarded it as a case with facial weakness, regardless of incomplete or complete paralysis. The time-points for facial nerve functions were postoperative day 1 to 5 and 6 to12 months. The facial weakness of one or more facial subsites at 6 to12 months after parotidectomy was defined as permanent facial palsy in this study. The patients with facial palsy, but with the intact anatomical facial nerve continuity were followed up to 12 months. However, when the facial nerve was sacrificed at the time of parotidectomy, it was diagnosed as permanent facial palsy at postoperative 6 months.

### Statistical analysis

The occurrence of facial weakness in each category was presented with descriptive statistics. To analyze risk factors for facial nerve weakness, multivariable logistic regression analysis was performed. Using the variables with a significant *P* value (less than 0.05) in univariable analyses, we generated a multivariable model, in which final estimates for each would yield an adjusted odds ratio for each factor retained in the model.

The potential associations of variables in multivariable analyses were evaluated calculating variance inflation factor for multicollinearity [45]. In addition, clinically suspected correlations of variables (e.g., tumor subsites versus extent of surgery) were reexamined with Pearson’s chi-square test, Fisher’s exact test, and Wilcoxon rank sum test as appropriate.

All *P*-values are two-sided, and a level of 5% was considered to be statistically significant. Statistical analysis was executed using SAS version 9.4 (SAS Institute, Cary, NC) and SPSS version 24 (IBM Co., Armonk, NY, USA).

## Results

### Subject characteristics

A total of 794 patients were included in this study (Table 1). The histopathology revealed that 651 patients had benign tumors (82.0%) and 143 patients had malignant tumors (18.0%) (Additional file 2: Table S1).

**Table 1:** Subject characteristics and facial nerve functional outcomes

No. (%)	Total patients (*n* = 794)	Facial weakness	
		Temporary^a^ (*n* = 73, 9.2%)	Permanent^b^ (*n* = 41, 5.2%)
Gender			
Male	392 (49.4%)	32 (8.2%)	16 (4.1%)
Female	402 (50.6%)	41 (10.2%)	25 (6.2%)
Age (years, mean, range)	48.9 [11–90]	52.4 [18–83]	54.4 [25–83]
Pathology (Supplementary Table S1)			
Benign tumors	651 (82.0%)	42 (6.5%)	22 (3.4%)
Malignant tumors^c^	143 (18.0%)	31 (21.7%)	19 (14.4%)
Tumor size (longest diameter, cm, mean, range)	2.6 [0.4–9.5]	3.0 [0.7–7.0]	3.1 [1.0–7.0]
Number of tumors			
Single	759 (95.6%)	67 (8.8%)	37 (4.9%)
Multiple (≥ 2)	35 (4.4%)	6 (17.1%)	4 (11.4%)
Tumor subsite			
Superficial to the facial nerve	667 (84.0%)	44 (6.6%)	26 (3.9%)
Deep to the facial nerve	70 (8.8%)	12 (17.1%)	4 (5.7%)
Superficial and deep location (both)	57 (7.2%)	17 (29.8%)	11 (19.3%)
Extent of surgery			
Extracapsular dissection of tumors	78 (9.8%)	2 (2.6%)	1 (1.3%)
Partial parotidectomy	441 (55.5%)	29 (6.6%)	12 (2.7%)
Superficial parotidectomy	187 (23.6%)	13 (7.0%)	10 (5.3%)
Total parotidectomy	88 (11.1%)	29 (33.0%)	18 (20.5%)
Types of surgery			
Primary parotidectomy	753 (94.8%)	62 (8.2%)	33 (4.4%)
Revision parotidectomy (Recurrent tumors)	41 (5.2%)	11 (26.8%)	8 (19.5%)

^a^Temporary facial weakness: Status of facial expression at postoperative day 1 to 5

^b^Permanent facial weakness: Status of facial expression at more than 6 months postoperatively

^c^Malignant tumors: facial nerve sacrifices in 21 cases with malignant tumors, even with intact facial nerve function pre-operatively

Among total patients, 73 patients (9.2%) had temporary facial weakness and 41 patients (5.2%) had permanent facial weakness. Meanwhile, the rates of temporary and permanent facial weakness were 21.7 and 14.4% for malignancy, 29.8 and 19.3% for tumors located in both superficial and deep lobes, 33.0 and 20.5% for cases with total parotidectomy, and 26.8 and 19.5% for revision surgery. In 21 patients out of the 143 malignant tumors, some branches of the facial nerve were intentionally sacrificed to secure the safety margin during surgery. Seven of the 21 patients of whom the buccal and cervical branches of the facial nerve had been sacrificed during surgery, had no facial weakness at 6 months postoperatively.

### Degree of facial dysfunction

Based on the House-Brackmann grading system, incomplete (grade 4 or less) temporary facial weakness was observed in 51 patients, and complete facial nerve palsy was observed in 22 patients, respectively (Table 2). At more than 6 months after parotidectomy, 25 patients still suffered from incomplete facial nerve palsy and 16 had complete facial nerve palsy. Regarding the subsites of facial expression, lip deviation was the most frequently affected subsite in both temporary and permanent facial weakness.

**Table 2:** Degree and extent of facial weakness

No.(%) House-Brackmann grading system	Facial weakness	
	Temporary^a^ (*n* = 73)	Permanent^b^ (*n* = 41)
Incomplete facial nerve weakness (grade ≤ 4)	51 (69.9%)	25 (61.0%)
Complete facial nerve weakness (grade 5–6)	22 (30.1%)	16 (39.0%)
Subsites (numbers overlapped)		
Forehead wrinkling	22 (30.1%)	19 (46.3%)
Eye closure	26 (35.6%)	18 (43.9%)
Lip deviation	73 (100%)	40 (97.6%)

^a^Temporary facial weakness: Status of facial expression at postoperative day 1 to 5

^b^Permanent facial weakness: Status of facial expression at more than 6 months postoperatively

### Analysis of risk factors for postoperative facial weakness

The results of univariable analysis showed that age, malignant tumor, total parotidectomy, recurrent tumor (revision parotid surgery), tumor size, and tumors subsites being deeper than the facial nerve and crossing the nerve (i.e., located in both superficial and deep lobes) were statistically significant risk factors for temporary facial weakness (*P* < 0.05) (Table 3). In the case of permanent facial weakness, tumors located only deeper than the facial nerve plane were no longer a risk factor, in contrast to temporary facial weakness (*P* < 0.05) (Table 4).

**Table 3:** Regression analyses of risk factors for temporary facial weakness after parotidectomy

Variables	Categories	Univariable				Multivariable			
		Odds Ratio	95% Confidence interval		*P* value	Odds Ratio	95% Confidence interval		*P* value
Gender	M vs. F (ref)	1.278	0.787	2.076	0.322				
Age	Continuous	1.019	1.002	1.036	0.032	1.019	1.001	1.037	0.035
Pathology	Malignancy vs. benign (ref)	4.013	2.420	6.656	< 0.0001	3.472	2.018	5.974	< 0.0001
Tumor size	Continuous	1.282	1.086	1.514	0.0033	0.139	0.949	1.368	0.162
Number of tumors	Multiple vs. single (ref)	2.137	0.857	5.331	0.103	1.539	0.534	4.437	0.424
Tumor subsite	Superficial (ref)	1				1			
	vs. deep	2.929	1.465	5.856	0.002	2.202	1.052	4.608	0.036
	vs. both	6.018	3.159	11.465	< 0.0001	3.335	1.600	6.950	0.001
Extent of surgery	ECD (ref)	1							
	vs. partial	2.675	0.625	11.444	0.185				
	vs. superficial	2.839	0.625	12.889	0.176				
	vs. total parotidectomy	18.678	4.283	81.455	< 0.0001				
Type of surgery (recurrent tumors)	Revision vs. primary surgery (ref)	4.087	1.953	8.549	0.0002	3.222	1.285	8.076	0.013

Extent of surgery was significantly correlated with tumor pathology (*P* < 0.001) and tumor subsite (*P* < 0.001) by Pearson’s chi-square test. Thus, the extent of surgery variable was excluded in these multivariable analyses. *ref* reference, *ECD* extracapsular dissection of tumors

**Table 4:** Regression analyses of risk factors for permanent facial weakness after parotidectomy

Variables	Categories	Univariable				Multivariable			
		Odds Ratio	95% Confidence interval		*P* value	Odds Ratio	95% Confidence interval		*P* value
Gender	M vs. F (ref)	1.558	0.819	2.966	0.177				
Age	Continuous	1.029	1.006	1.053	0.013	1.031	1.007	1.055	0.011
Pathology	Malignancy vs. benign (ref)	4.381	2.302	8.336	<0.0001	4.019	2.006	8.092	<0.0001
Tumor size	Continuous	1.316	1.074	1.613	0.008	1.159	0.932	1.441	0.186
Number of tumors	Multiple vs. single (ref)	2.518	0.844	7.507	0.098				
Tumor subsite	Superficial (ref)	1				1			
	vs. deep	1.494	0.506	4.412	0.467	0.972	0.309	3.056	0.961
	vs. both	5.895	2.741	12.680	<0.0001	2.769	1.130	6.788	0.026
Extent of surgery	ECD (ref)	1							
	vs. partial	2.154	0.276	16.804	0.464				
	vs. superficial	4.350	0.547	34.578	0.165				
	vs. total parotidectomy	19.800	2.576	152.201	0.004				
Type of surgery (recurrent tumors)	Revision vs. primary surgery (ref)	5.289	2.266	12.345	0.0001	4.270	1.483	12.290	0.007

Extent of surgery was significantly correlated with tumor pathology (*P* < 0.001) and tumor subsite (*P* < 0.001) by Pearson’s chi-square test. Thus, the extent of surgery variable was excluded in these multivariable analyses. *ref* reference, *ECD* extracapsular dissection of tumors.

When conducting multivariable analysis, we first checked the multicollinearity and the potential correlations between variables (Additional file 3: Table S2). Variance inflation factors of variables were less than 2.0, suggesting that each variable had no significant effect within the multivariable regression model, based on the collinearity of variables [45]. However, the extent of surgery was significantly bigger in cases with malignant tumors (*P* < 0.001) and in cases with deep lobe tumor or tumor crossing the nerve (*P* < 0.001), according to Pearson’s chi-square test. Thus, the extent of surgery variable was excluded in these multivariable analyses. The remaining variables within a multivariable model showed variance inflation factors of less than 2.0.

Multivariable analysis revealed that age, malignancy, and recurrent tumors were common independent risk factors for both temporary and permanent postoperative facial weakness (*P* < 0.05). In addition, tumor subsite was associated with postoperative facial weakness. Particularly, a tumor subsite of the deep lobe was a risk factor for temporary facial weakness, but not for permanent facial palsy. Meanwhile, a tumor involving both superficial and deep lobe (crossing the facial nerve plane) was a significant negative factor for temporary and permanent facial weakness (Tables 3 and 4). Tumor size itself was not an independent risk factor for postoperative facial weakness.

## Discussion

Facial weakness is a major complication after parotidectomy, which severely affects patient quality of life postoperatively. The reported incidences ranges from 14.0 to 23.1% in terms of temporary facial weakness, [15, 18, 23, 25] with one exceptionally high occurrence of 64.6% [21]. Even with intraoperative facial nerve monitoring, the frequency of temporary facial weakness was within a similar range (20.0–33.3%) [13, 14, 17, 25]; meanwhile, permanent facial weakness was less frequently seen, ranging from 0.0 to 9.0% after parotidectomy [13, 14, 17, 19, 25]. In contrast, one meta-analysis reported that intra-operative facial nerve monitoring could not decrease the rate of permanent facial weakness in primary parotidectomies [35].

To reduce facial nerve complications of parotidectomy, several techniques have been applied including preoperative imaging and intraoperative facial nerve monitoring. However, the surgical outcomes using these techniques have not been fully studied except for a few studies [10, 13, 14, 27]. In this study, we uniformly applied preoperative CT scans, intraoperative facial nerve monitoring and surgical magnifications among our patients. Although the preoperative CT and intraoperative facial nerve monitoring had some limitations in diagnosis, prediction, application and interpretation in practice, we found that the occurrence of temporary facial weakness in our cases (9.2%) was less than in previous studies, with a similar incidence of permanent facial weakness (5.2%). This may be due to a cautious, meticulous dissection of tumors around the facial nerve with the aid of these techniques. However, the incidence of permanent facial weakness was not different significantly following our technical assistances from the previous studies [13, 14, 17, 19, 25]. Thus, a tumor factor rather than surgical technique may be a major determinant of permanent facial weakness. We think that intra-operative facial nerve monitoring combined with proper surgical planning and surgical magnification could decrease the facial nerve injury by technical problems (temporary facial palsy).

There have been many studies to date that have analyzed risk factors for facial paralysis/palsy after parotidectomy. Among variables, old age, malignancy, tumor size, operation time, revision surgery and extent of surgery were reported as risk factors for postoperative facial weakness [12, 14–17, 19, 22, 23, 27, 30, 46]. In this study, we defined eight parameters for potential risk factors for facial weakness, which were gender, age, tumor pathology (malignancy), size, number, and subsite (location), the extent of surgery and revision surgery. In concordance with previous reports, we found that old age, malignant tumor, and recurrent tumor were independent risk factors for both temporary and permanent postoperative facial weakness. In addition, we found that tumor subsite was significantly associated with postoperative facial weakness and tumor size itself was not an independent risk factor for postoperative facial weakness.

Regarding the age of patients, there have been inconsistent results in the previous literature [15, 18, 19]. However, it is generally accepted that recovery after facial nerve injury or regeneration is slower in elderly patients versus younger patients [47]. Malignancy and recurrent tumors were common risk factors of postoperative facial weakness in previous studies [14–17]. In the case of malignant tumors, it is important to secure a safety margin during surgery. Therefore, there can be a high possibility that the facial nerve is intentionally resected during surgery. In this study, 21 of 143 malignant tumors were sacrificed for safe resection.

In our study, the size of the tumor was not a significant risk factor for facial weakness, but the subsite of the tumor was. Previously, one study found that tumors larger than 70 cm^3^ correlated with a significant risk for facial weakness [15]. Another study indicated that the greater the tumor size is, the higher the chance of permanent facial weakness was with an odds ratio of 2.66 [30]. However, a tumor subsite (location) was not considered in those studies as variables [15, 30]. Rather, the location of the tumor has been reported as a significant risk factor for facial weakness in a previous study [17], supporting our findings.

In the present study, preoperative CT allowed us to estimate the positional relationship between the imaginary facial nerve line and the tumor before surgery. However, this indirect method on CT images showed only a modest diagnostic accuracy for localization of parotid tumors in relation to the facial nerve [37]. Thus, the tumor subsites in this study were classified based on the surgical findings, not on the CT images. Recent advanced techniques of magnetic resonance imaging may have clinical benefit of more accurate surgical planning by directly visualizing the facial nerve in the parotid gland [37].

With the aid of intraoperative nerve monitoring and surgical magnification, the anatomical continuity of the facial nerve could be preserved, even with rerouting of the facial nerve during surgery for tumors located in both the superficial and deep lobes. Although temporary facial weakness might be caused by excessive manipulation of the facial nerve, rates of permanent facial weakness seemed not to be high due to preservation of the nerve continuity and recovery of the facial nerve conduction.

Although our study provided clinical information about the incidence and risk factors of facial weakness in parotidectomy using currently available tools, the results should be further validated in prospective comparative studies. This is of note because our data are collected retrospectively, and there may be incomplete clinical information, selection bias and unnoticed confounders. Also, the cohort of this study was heterogeneous, including both primary and revision surgery, both benign and malignant tumors. Therefore, additional studies on specific patient groups will be needed in the future. In addition, the responsible surgeons were all experienced and skilled in our study, and surgical preferences among surgeons can be different. Thus, our results cannot be directly extrapolated to general patients receiving parotidectomy.

In spite of these limitations, we found that the tumor subsite was a risk factor for postoperative facial weakness in parotidectomy in addition to the known risk factors. This can be overcome by meticulous surgical techniques, in collaboration with the preoperative imaging study, intraoperative facial nerve monitoring, and surgical magnifications.

## Conclusion

Overall incidences of temporary and permanent (more than 6 months) facial weakness were 9.2 and 5.2% in our series utilizing preoperative CT, intraoperative facial nerve monitoring, and surgical magnification. In addition to the known risk factors for facial weakness in parotid tumor surgery (e.g., old age, malignant or recurrent tumors), the tumor subsite was found to be related to postoperative facial weakness. Thus, preoperative counseling of the patients with risk factors and delicate manipulation of the facial nerve seems to be necessary to reduce facial nerve complications.

## Supplementary information

**Additional file 1..** Raw data.

**Additional file 2. Table S1.** Pathology of subjects.

**Additional file 3. Table S2.** Multicollinearity of variables in multivariable risk factor analyses for facial weakness.

## Data Availability

All data generated or analyzed during this study are included in this published article and its supplementary information file (Additional file 1. Raw data).

## Abbreviations

CT: Computed tomography
ECD: Extracapsular dissection of tumors
MRI: Magnetic resonance imaging
