# Supplementary material for: Incidence of postoperative facial weakness in parotid tumor surgery: a tumor subsite analysis of 794 parotidectomies
Source: BMC Surg. 2019 Dec 26;19:199. doi: 10.1186/s12893-019-0666-6 (PMC6933669; doi:10.1186/s12893-019-0666-6)
Supplement: Supplementary file 2 — Additional file 2. Table S1. Pathology of subjects. [file 12893_2019_666_MOESM2_ESM.docx]

**Table S1.** Pathology of subjects

| **No. (%)** | | **Total patients (n = 794)** |
| --- | --- | --- |
| Benign tumors | | 651 (82.0%) |
|  | Pleomorphic adenoma | 413 (52.0%) |
|  | Warthin tumor | 120 (15.1%) |
|  | Basal cell adenoma | 39 (4.9%) |
|  | Lymphoepithelial cyst | 12 (1.5%) |
|  | Myoepithelioma | 11 (1.4%) |
|  | Chronic recurrent inflammation | 10 (1.3%) |
|  | Lymphadenitis (bacterial) | 9 (1.1%) |
|  | Oncocytoma | 6 (0.8%) |
|  | Neurogenic tumor | 5 (0.6%) |
|  | Lipoma | 4 (0.5%) |
|  | Kimura’s disease | 3 (0.4%) |
|  | Cystadenoma | 3 (0.4%) |
|  | Lympho-venous malformation | 3 (0.4%) |
|  | Monomorphic adenoma | 2 (0.3%) |
|  | Tuberculosis lymphadenitis | 2 (0.3%) |
|  | Retention cyst | 2 (0.3%) |
|  | Others | 7 (8.8%) |
| Malignant tumors | | 143 (18.0%) |
|  | Mucoepidermoid carcinoma | 33 (4.2%) |
|  | Salivary ductal carcinoma | 20 (2.5%) |
|  | Lymphoma | 18 (2.3%) |
|  | Acinic cell carcinoma | 16 (2.0%) |
|  | Adenoid cystic carcinoma | 16 (2.0%) |
|  | Carcinoma ex pleomorphic adenoma | 9 (1.1%) |
|  | Adenocarcinoma, not otherwise specified | 7 (0.9%) |
|  | Basal cell adenocarcinoma | 6 (0.8%) |
|  | Mammary analogue secretory carcinoma | 5 (0.6%) |
|  | Squamous cell carcinoma | 5 (0.6%) |
|  | Lymphoepithelial carcinoma | 2 (0.5%) |
|  | Epithelial myoepithelial carcinoma | 2 (0.3%) |
|  | Myoepithelial carcinoma | 2 (0.3%) |
|  | Oncocytic carcinoma | 1 (0.1%) |
|  | Cystadenocarcinoma | 1 (0.1%) |
