# Supplementary material for: Incidence of postoperative facial weakness in parotid tumor surgery: a tumor subsite analysis of 794 parotidectomies
Source: BMC Surg. 2019 Dec 26;19:199. doi: 10.1186/s12893-019-0666-6 (PMC6933669; doi:10.1186/s12893-019-0666-6)
Supplement: Supplementary file 3 — Additional file 3. Table S2. Multicollinearity of variables in multivariable risk factor analyses for facial weakness. [file 12893_2019_666_MOESM3_ESM.docx]

**Table S2.** Multicollinearity of variables in multivariable risk factor analyses for facial weakness

| **Variables** | **Variance inflation factor** | | |
| --- | --- | --- | --- |
|  | **Temporary facial weakness** | **Permanent facial weakness** | |
| Gender | - | 1.056 |  |
| Age | 1.018 | 1.023 |  |
| Pathology | 1.035 | 1.001 |  |
| Tumor size | 1.080 | 1.052 |  |
| Number of tumors^a^ | 1.114 | - |  |
| Tumor subsite^b^ | 1.132 | - |  |
| Types of surgery^c^ | 1.152 | 1.010 |  |

^a^Number =1 vs. ≥ 2
^b^Superficial to the facial nerve vs. deep to the facial nerve vs. both

^c^Primary vs. revision surgery
